# An integrated commercial poultry breeding framework for natural mating and animal welfare

**DOI:** 10.1016/j.psj.2026.107938

**Published:** 2026-10-10

**Authors:** Zhuo-Cheng Hou

**Affiliations:** Frontier Science Center for Molecular Design Breeding, National Engineering Laboratory for Animal Breeding and Key Laboratory of Animal Genetics, Breeding and Reproduction, Ministry of Agriculture and Rural Affairs, College of Animal Science and Technology, China Agricultural University, Beijing, 100193, China

**Keywords:** Poultry breeding, Free-range, Genomic selection, Natural mating, Precision livestock farming

## Abstract

Current breeding programs were designed for intensive cage-based production and cannot fully support future welfare-oriented poultry systems. This review proposes a novel commercial poultry breeding framework explicitly designed for natural mating and free-range environments. The proposed system integrates several major interconnected core pillars: (1) fully automated phenotyping utilizing 3D computer vision, radio-frequency identification (RFID), and Internet of Things (IoT) sensors to continuously collect individual-level behavioral and/or physiological data in open environments; (2) genome-based pedigree reconstruction to accurately infer parent-offspring relationships when natural mating precludes controlled artificial insemination; (3) genomic best linear unbiased prediction (GBLUP) for precise breeding value estimation in large, randomly mating populations; (4) natural mating without predetermined mate allocation in large populations; and (5) multi-omics integration accounting for gene-environment interactions to enhance climate resilience. These pillars operate synergistically: automated phenotyping and genomic pedigree tracking generate high-density data streams that feed into genomic prediction models, while multi-omics approaches refine selection decisions under variable environmental conditions. Unlike conventional cage-based systems that rely on artificial insemination, this framework is designed to eliminate labor associated with artificial insemination and enables integration of pedigreed pure lines and great-grandparent populations. Conditional on active genomic management of reproductive skew, it has the potential to sustain high genetic diversity within large natural-mating cohorts, thereby offering prospects for reduced long-term operational costs alongside improved environmental adaptability and bird welfare. Key transition challenges include high initial capital investment in automated phenotyping infrastructure and the prerequisite for robust genomic selection pipelines. To our knowledge, this is the first review proposing an integrated commercial breeding framework specifically designed for free-range natural mating systems.

## Introduction

As one of the fastest-growing sectors in global agriculture, the poultry industry serves as a highly efficient and cost-effective source of animal protein for human consumption (Mottet and Tempio, 2017). Annual global egg production reached approximately 97 million tonnes in 2023, while more than 100 billion chickens are slaughtered annually for meat production worldwide (FAO, 2023). Global demand for poultry meat has continued to increase owing to its affordability, nutritional value, and production efficiency (Nkukwana, 2019), and poultry is projected to surpass pork as the world's most widely consumed meat by 2027 (OECD/FAO, 2024). Decades of intensive genetic selection have enabled modern commercial broilers to reach market weight within 5–6 weeks while achieving exceptional feed efficiency, making poultry one of the most resource-efficient terrestrial livestock production systems. Furthermore, the spatial scalability of poultry husbandry enables successful production across diverse environments, including small-scale or land-constrained smallholder farms. These biological and economic efficiencies render poultry production an essential driver of food security and economic viability, particularly within developing countries and emerging markets.

Genetic selection has historically been the cornerstone of poultry industry advancements, contributing approximately 50% to cumulative performance gains over the past several decades (Wolc, 2015, 2016; Zhang, et al., 2020). It was the groundbreaking work by Meuwissen, et al. (2001) on genomic selection (GS)–predicting total genetic value using genome-wide dense marker maps–that fundamentally transformed animal breeding. The integration of genomic relationship matrices into BLUP frameworks through genomic BLUP or variants (single-step GBLUP, ssGBLUP; Legarra, et al., 2009; GBLUP, VanRaden, 2008) has since enabled routine genomic prediction in commercial poultry breeding (Zhang, et al., 2020). Nevertheless, at the elite (pure lines) breeding tier—where genetic improvement is actually generated—these methodological innovations have been predominantly developed and validated within intensively managed systems with controlled environments, artificial insemination (AI), and complete pedigree recording. This characterization applies broadly to both layer and broiler elite programs, although their operational details differ: broiler elite lines are typically maintained in floor or cage pens with individually pedigreed matings, whereas layer elite programs have historically relied heavily on cage-based AI schemes. Importantly, natural mating is already standard practice downstream of the elite tier: commercial broiler production is multiplied almost exclusively through natural mating in floor-housed parent-stock flocks, and natural mating is also used at multiplication levels in layer production. The framework proposed here therefore does not introduce natural mating to poultry production as such; rather, it extends natural mating into the elite breeding tier and couples it with the genomic pedigree reconstruction and automated phenotyping required to sustain accurate selection without controlled mating.

This methodological optimization for intensive systems, however, faces mounting challenges as the industry confronts shifting societal expectations and regulatory landscapes. Consumer preferences and legislative frameworks in numerous jurisdictions–including the European Union, several U.S. states, and Australasia–increasingly mandate transitions away from conventional cage-rearing systems due to welfare concerns regarding confinement stress, skeletal injury, feather pecking, and associated mortality (de Almeida and Zuber, 2010; Larsen, et al., 2018). The European Union’s phased prohibition of conventional battery cages exemplifies this regulatory momentum (Taylor, et al., 2018). Empirical assessments demonstrate that hens in cage-free systems exhibit greater behavioural expression–including foraging, dust-bathing, and perching–though these benefits must be balanced against potential increases in predation risk (Bonnefous, et al., 2022). These evolving pressures are fundamentally reshaping the production environment in which breeding objectives must be realized.

Concurrently, accelerating climate variability and the increasing frequency of extreme weather events necessitate the development of robust, climate-resilient genotypes capable of maintaining production outside climate-controlled housing (Nardone, et al., 2010). Heat stress alone costs the global poultry industry billions of dollars annually through reduced feed intake, compromised immunity, and elevated mortality (Renaudeau, et al., 2012). The genetic architecture of heat tolerance–involving polygenic networks governing feather coverage, vascularization, and hypothalamic-pituitary-adrenal axis regulation–presents both a challenge and an opportunity for genomic selection targeting environmental adaptability (Hill, 2016). The capacity to select for thermotolerance without sacrificing production efficiency has become an increasingly urgent breeding priority.

Compounding these biological challenges, escalating labor costs and the logistical difficulty of error-free pedigree recording in large-scale operations are accelerating the shift toward automated, minimally staffed husbandry systems (Astill, et al., 2020). Precision livestock farming (PLF) technologies–including computer vision-based monitoring, wearable sensors, and automated weighing–offer high-throughput phenotyping opportunities in loose-housing environments (Neethirajan, 2022). These advances reduce dependence on labor-intensive artificial insemination frameworks, signalling a structural transition toward automated breeding operations and creating both the necessity and foundation for reimagining poultry breeding within alternative production systems.

Collectively, these shifting paradigms require poultry breeding programs to optimize multiple breeding objectives, including production efficiency, environmental adaptability, and high standards of animal welfare. To bridge the gap between conventional intensive breeding systems and emerging sustainable production systems, this paper proposes an integrated commercial poultry breeding framework specifically designed for free-range farming and natural mating environments. By leveraging advances in precision livestock farming (PLF), automated phenotyping, population genomics, and multi-omics data integration, the proposed framework combines genomic selection, automated pedigree reconstruction, high-throughput phenotyping, and digital technologies to support natural mating, loose-housing production systems, and climate resilience within a commercial breeding context. Rather than replacing existing breeding systems, this framework provides a complementary strategy for developing welfare-oriented, environmentally resilient, and genetically sustainable poultry breeding programs.

## Framework of the proposed breeding system

The proposed framework is termed the Natural Mating and Animal Welfare Poultry Breeding System (NMAW-PBS). This system operates without pre-determined, restrictive individual male-to-female mating plans. Instead, birds move freely within loose housing or free-range environments and mate naturally within a large population cohort. By deploying high-throughput individual identification, automated phenotypic tracking, and genomic sequencing, individual genomic estimated breeding values (GEBVs) can be accurately calculated to drive selection decisions for subsequent generations. The proposed breeding system is displayed in Fig. 1.

**Fig. 1:**
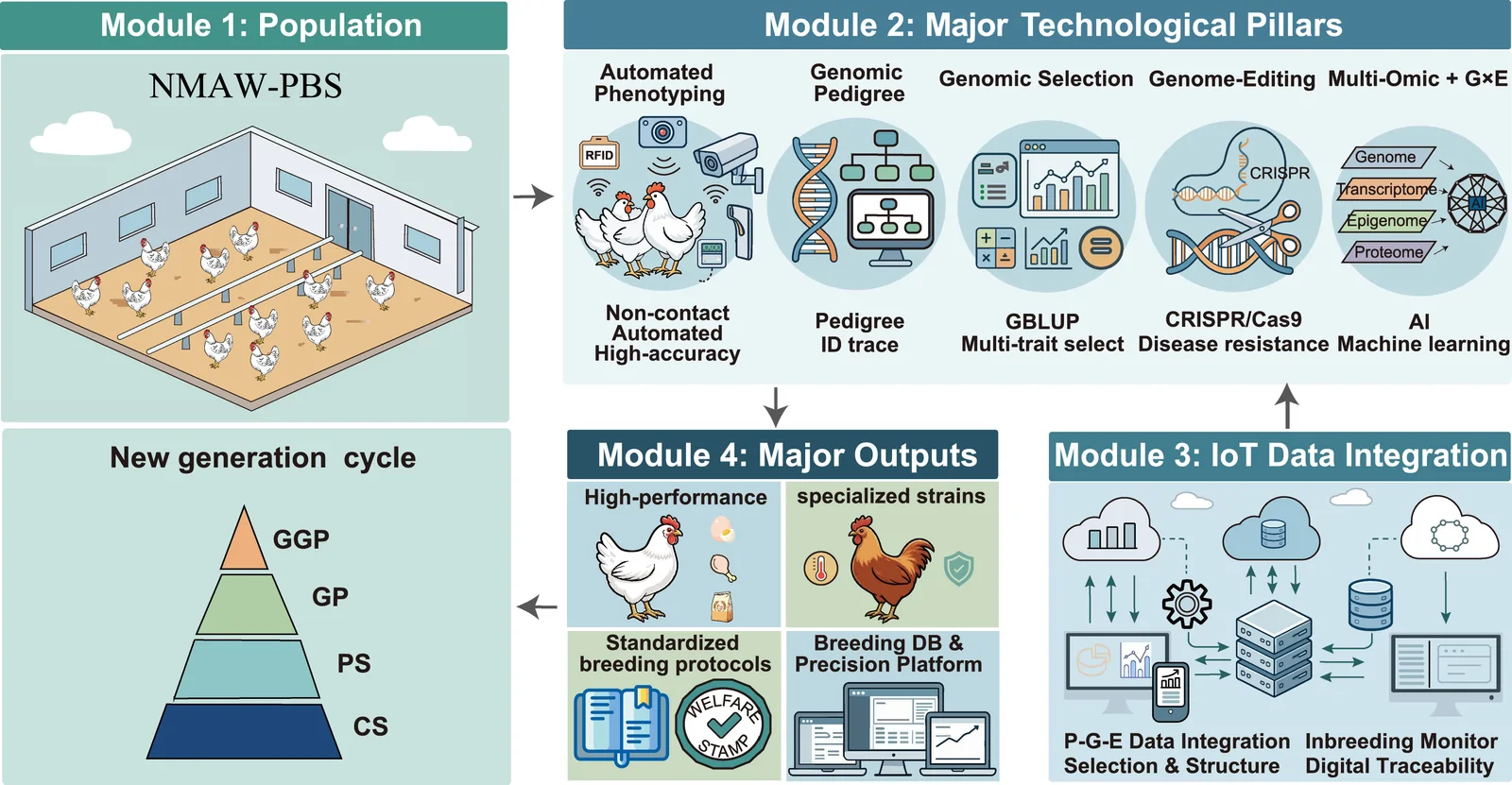
Conceptual framework of an integrated poultry breeding system for natural mating and animal welfare. The framework includes four modules: (1) Breeding Population, featuring natural mating in open free-range environments and animal welfare adapted; (2) Major Technological Pillars, including automated phenotyping, genomic pedigree reconstruction, genomic selection, genome editing, and environmental monitoring; (3) IoT Data Integration, which combines edge computing and digital twin technology within a unified IoT architecture to collect and integrate phenotypic, genomic, and environmental data in real time; and (4) Output, selected candidates to produce the next generation production. Solid arrows indicate data flow, and dashed arrows indicate feedback loops. Abbreviations: NMAW-PBS = Natural Mating and Animal Welfare Poultry Breeding System; GEBV = genomic estimated breeding value; IoT = Internet of Things; RFID = radio-frequency identification; SNP = single nucleotide polymorphism; WGS = whole-genome sequencing; GBLUP = genomic best linear unbiased prediction; ssGBLUP = single-step genomic best linear unbiased prediction; IBD = identity-by-descent.

The most significant difference between the NMAW-PBS and the family-based breeding system is that the NMAW-PBS does not require a clear family structure within the breeding population. All birds are allowed to move freely and mate naturally—without predetermined mate allocation—in a large, open rearing environment. Since the NMAW-PBS allows pure lines to be maintained in much larger populations than conventional nucleus schemes, the breeding tier can, under appropriate conditions, be integrated with GGP multiplication. Natural mating is explicitly not equated with random mating in this framework (see 'Potential genetic risks under unrestricted natural mating'): social dominance, mate preference, and competition generate reproductive skew, so the realized mating structure must be monitored and managed rather than assumed. Phenotypic records for most traits are collected continuously for each bird in the pure lines population under open rearing conditions, while genotypes can be obtained for any individual as needed. Genomic estimated breeding values (GEBVs) are estimated for all birds, and elite individuals are selected for the next generation based on these GEBVs.

A major challenge is locating and retrieving the selected birds from the large elite population. Precision livestock farming technologies, such as individual egg tracking, can be used to trace and collect eggs from specific hens. In practice, the pure line population may be subdivided into several large subgroups housed within the same breeding facility, which facilitates identification of the selected individuals. For meat-type breeding programs, progeny performance data can also be used to refine parental breeding values. The application of genomic mating to produce the next generation presents another challenge in the NMAW-PBS system. However, this approach also offers an opportunity to generate more genetic combinations than are practically achievable with family-based breeding tactics. Once the selected birds are identified, they are preferably captured and transferred to a parallel Breeding/GGP farm for dedicated rearing of the next generation. Therefore, a minimum of two Breeding/GGP farms is required simultaneously—one designated for breeding and another serving as a backup—to sustain both the breeding cycle, elite individual backup, and the GGP production cycle.

The core structural pillars of this system comprise:

(1) On-site automated phenotyping: Continuous recording of major individual phenotypes–including body weight, feed consumption, egg production, and health status–via automated field devices.
(2) Genomic pedigree reconstruction: Resolving individual kinship in large population and constructing accurate pedigree structures using whole-genome sequence data or high-density single nucleotide polymorphism (SNP) chips.
(3) Breeding value estimation: Utilizing GBLUP or ssGBLUP as the primary computational engines to derive GEBVs. All candidates in the breeding population should be genotyped for estimating GEBVs.
(4) Natural mating dynamics: Allowing individuals to range freely and express natural mating behaviors within large group-housed populations. Operational parameters for natural mating within this framework are designed to balance genetic diversity, mating efficiency, and animal welfare.
(5) Integrated data architecture: Synchronizing Internet of Things (IoT) sensors with existing breeding data platforms through automated big data analysis pipelines.

Within the NMAW-PBS, the realized mating structure is reconstructed empirically each generation rather than assumed. Key genetic metrics including realized effective population size *N_e_*, reproductive skew, and runs of homozygosity (ROH) are monitored routinely. When reproductive skew exceeds predefined thresholds—for example, when a small fraction of sires produces a disproportionate share of progeny, or when realized *N_e_* falls below the target range—corrective management is applied. Such measures include rotation or removal of excessively dominant males, rebalancing of sex ratios, subdivision of very large groups into multiple mating cohorts to reduce resource monopolization. In this way, the welfare benefits of natural mating behavior are preserved while its genetic consequences are kept within acceptable limits.

The structural parameters and differences between current proposed NMAW-PBS and the traditional model are explicitly contrasted in Table 1.

**Table 1:** Comparative overview of conventional breeding systems between NMAW-PBS.

Main Parameter	Conventional Breeding System	NMAW-PBS
Rearing Pattern	Individual or family cages; closed, climate-controlled housing environments	Free-range; unconstrained open loose-floor layouts
Animal Welfare Index	Spatial constraints in cage systems; controlled handling protocols; welfare concerns driving transition to enriched and cage-free alternatives	Free movement; group social interactions; minimized contact-stress environment
Breeding Value Estimation	Pedigree BLUP (PBLUP), GBLUP or variants	Full GBLUP or variants
Family Verification	Requires highly restrictive, manual mating allocations	Automated individual relationship discovery via genome information
Mating Configuration	Artificial insemination prevalent in both layer and broiler breeding	Complete natural mating
Phenotype Collection	Variable automation; ranging from manual recording to semi-automated feeding and weighing stations; advanced systems used by major breeding companies	High-throughput automation: 3D vision systems, RFID, and multi-sensor IoT platforms
Environmental Control	Climate-controlled housing with artificial ventilation, lighting, and temperature regulation; high energy inputs.	Much less control on housing conditions; lower energy inputs but higher weather-dependent variability.
Genotyping Strategy	Variable or minimal; traditional selection focuses on phenotypes	Obligatory target genotyping using DNA chips or whole-genome sequencing
Pedigree Construction	Manual recording; strictly tracking isolated parental pairs	Fully depends on genome information
Inbreeding Management	Regulated exclusively by deliberate manual mating pairing selections	Proactive monitoring via genomic relationship matrices; inbreeding managed through algorithm-based optimal mating allocations post-genomic selection.
Individual and Environmental Interaction	Primarily managed through controlled housing; multi-site testing used to capture some GxE variation.	Deeply incorporated environment factors; climate resiliency selection.
Data Control Engine	Localized data management; isolated paper records or basic spreadsheets	Unified 3-layer IoT cloud architecture; edge-computing; digital twins
Effective Population Size	Moderate genetic base; maintained through multi-line crossing and structured selection programs	Potential higher effective population size through large-group natural mating
Primary Selection Aims	Production efficiency metrics (growth rate, feed conversion ratio, egg production)	Production, behavior, and climate resilience, with increasing inclusion of fitness, robustness, and welfare-related traits
Labor Requirements	Labor-intensive, particularly for artificial insemination in layer breeding and manual pedigree recording; variable across species and tiers	Lower manual field footprint; transitioned toward digital analytical systems (anticipated)
Cost Dynamics	High cage/facility infrastructure overheads; intense manual variable payroll	Higher upfront hardware capital costs; potentially lower long-term operational costs (awaiting large-scale commercial validation)
Disease Transmission Risk	Lower within-flock transmission risk due to physical separation; rapid containment possible through compartmentalization	Higher potential for horizontal transmission via social contacts and shared ranging areas; monitoring relies on digital health tracking
Selection Accuracy	Moderate to high accuracy depending on pedigree completeness and genomic data availability	Accuracy contingent on successful pedigree reconstruction and data pipeline integrity
Scalability	Well-established scaling protocols; large-scale commercial operations with proven multiplication factors across tiered pyramid structures	Still under evaluation for large-scale commercial deployment

**Abbreviations:** BLUP = best linear unbiased prediction; PBLUP = pedigree-based BLUP; GBLUP = genomic BLUP; ssGBLUP = single-step GBLUP; GGP = great grandparent; GP = grandparent; PS = parent stock; CS = commercial stock; IoT = Internet of Things; RFID = radio-frequency identification; 3D = three-dimensional; GS = genomic selection; GxE = genotype-by-environment; SNP = single nucleotide polymorphism.

### Operational workflow of the NMAW-PBS

To make the framework operational, its implementation follows an eight-step, generation-by-generation rotational cycle, which is schematically depicted together with indicative reference population benchmarks and key decision-making points in Fig. 2.

(1) Establishment of the breeding cohort. Each elite line is maintained within one or several large cohorts reared in loose-floor or free-range housing at GGP Farm A, which acts as the active breeding nucleus for the current generation.
(2) Hatching and genotyping. All newly-hatched chicks receive wing-bands or leg-mounted RFID tags at hatch. Tissue samples (wing-vein blood or feather pulp) are collected for genotyping via SNP chips or low-pass whole-genome sequencing (WGS).
(3) Automated phenotyping. RFID-enabled feeding and weighing stations, smart nest boxes, and 3D machine-vision systems continuously collect individual-level phenotypic and behavioural records across the rearing period.
(4) Candidate identification and genomic evaluation. GEBVs are iteratively updated as phenotypic data accumulate. For meat-type lines, initial selection can be performed around 6 weeks of age; for layers, evaluation occurs at sexual maturity and peak lay. The single-step genomic best linear unbiased prediction (ssGBLUP) model integrates genomic data, genomically-reconstructed pedigree information, and individual phenotypes. Optimal contribution selection (OCS) is subsequently applied to define parental candidates, maximizing expected genetic gain under predefined inbreeding-rate constraints.
(5) Retrieval of selected elite birds. Target animals are localized within the large mixed cohort using fixed and handheld RFID readers. Smart nest boxes identify laying hens and their respective eggs; RFID-gated catching corridors and trap-nest infrastructure support physical animal retrieval. Birds may also be captured during routine manual-handling events including immunization. Developing diverse practical approaches to facilitate reliable individual identification within large flocks is therefore essential. Non-selected individuals remain at Farm A, serving as a genetic reserve and continuing phenotypic recording to supply candidates for multiplication tiers.
(6) Transfer and natural mating. Pre-selected elite birds are physically transferred to the parallel backup GGP Farm B to constitute the reproductive cohort for the upcoming generation. No human-assigned mate allocations are implemented; animals mate naturally within this group-housed flock.
(7) Offspring production and individual identification. Eggs are collected over a standardized defined laying window. All hatched progeny are tagged and tissue-sampled for genotyping at hatch.
(8) Genomic parentage reconstruction, monitoring and generation turnover. SNP-based parentage assignment via identity-by-descent (IBD) analysis reconstructs sire-dam relationships for each offspring and validates realized mating patterns. Genomic parentage assignment of all offspring yields the realized progeny distribution of every sire and dam, from which variance in family size, realized effective population size, and genomic coancestry are computed routinely.

**Fig. 2:**
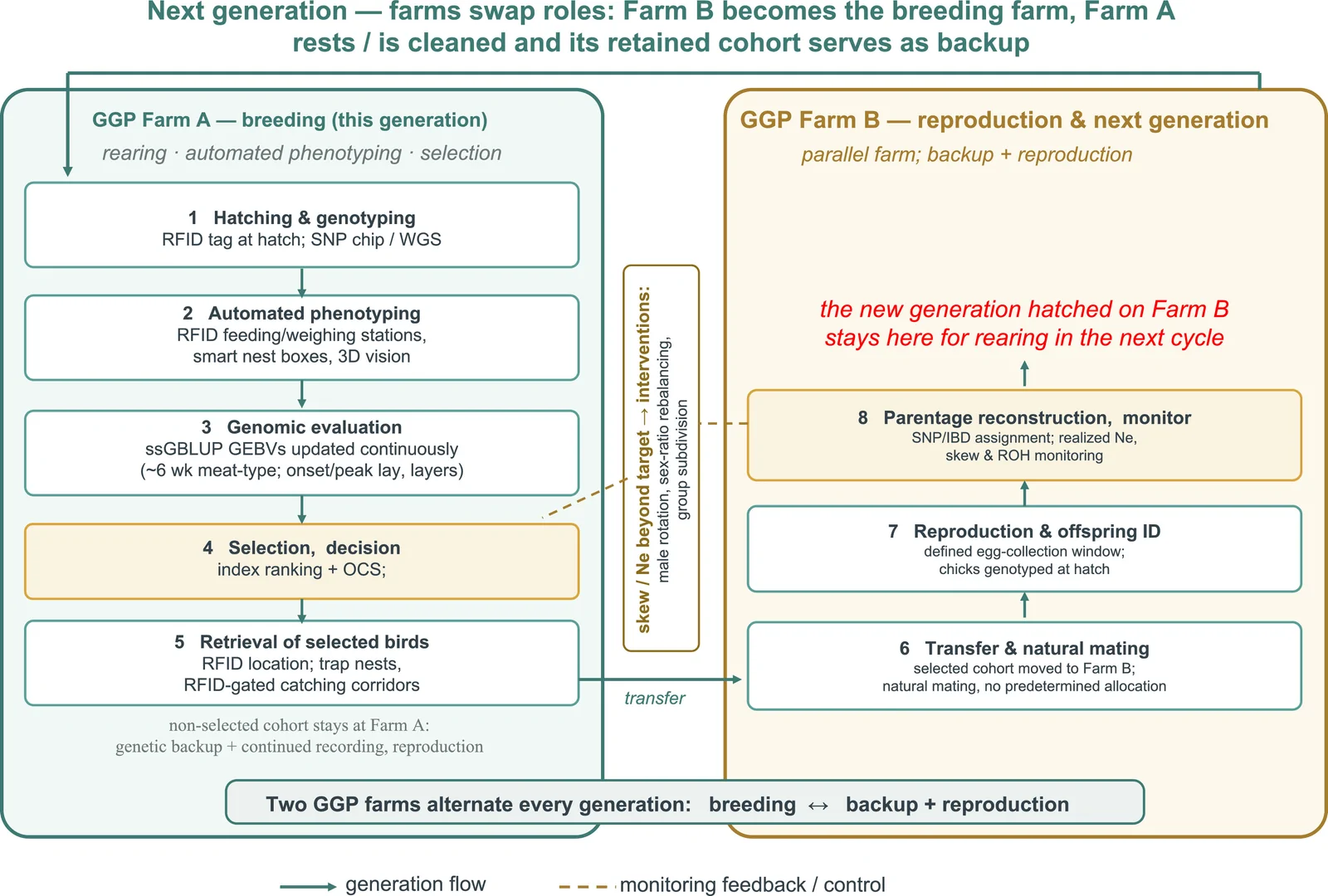
Generation-by-generation workflow of the NMAW-PBS with two-GGP-farm rotation. On the breeding farm (left, farm A): (1) chicks are RFID-tagged and genotyped at hatch, (2) phenotyped automatically; (3) Breeding evaluation, and (4) bird retrieved via RFID; (5) the non-selected cohort remains as genetic backup and reproduction population. Selected birds are transferred to the parallel farm (right, farm B) for: (6) Natural mating; (7) Offspring hatching and genotyping, and (8) SNP/IBD-based parentage reconstruction with monitoring of realized effective population size and reproductive skew. The two farms alternate between breeding and rest/cleaning plus backup duties each generation (solid return arrow); the dashed arrow indicates feedback interventions (male rotation, sex-ratio rebalancing, group subdivision) when skew or *N_e_* exceeds target thresholds.

Once the Farm A finished the first round selection and breeder transfer, then the newly-hatched generation is reared on Farm B. Meanwhile, Farm A undergoes full sanitation and a biosecurity resting period. The two facilities then functionally swap roles for the subsequent breeding cycle: Farm B becomes the active breeding nucleus and Farm A acts as the backup reproductive site. At least two independent GGP farms are mandatory to sustain this rotational workflow and enable biosecurity breaks between generations.

## System strengths and limitations

### Advantages

**(1) Enhanced Animal Welfare via Natural Mating, and Free-Ranging**: Eliminating artificial insemination is expected to reduce physiological stress, handling injuries, and associated health complications in breeding flocks. This benefit has not yet been quantified within the NMAW-PBS itself; it is inferred from welfare studies of handling and insemination procedures and should be validated under the proposed system. Avoiding multi-tier cages satisfies emerging global animal welfare mandates and allows for natural behavioral expression (Larsen, et al., 2018). Because commercial poultry multiplication relies predominantly on natural mating, selecting candidates under identical environmental dynamics ensures that genetic gains transfer effectively to commercial production tiers.

**(2) Streamlined Infrastructure and Accelerated Genetic Progress**: Integrating automated phenotyping with natural mating would allow pure-line selection to occur directly within large production cohorts. This configuration has the potential to compress the traditional multi-tier selection pyramid (pure line, great grand parent to commercial), thereby reducing fixed rearing costs and shortening the lag in genetic progress by up to one generation. We stress that our work conceptualizes this integrated breeding framework rather than presenting completed experimental or simulation validation. No quantitative empirical or simulation data are yet available to confirm the actual magnitude of cost reduction and genetic-lag shortening under NMAW-PBS; these metrics constitute key targets for follow-up simulation modelling and on-farm commercial trials. Furthermore, continuous tracking of individual metrics (e.g., feed intake, oviposition timing, weight flux) is expected to provide granular data to optimize flock health and selection accuracy (Peng, et al., 2022; Zhou, et al., 2023).

**(3) Simultaneous Pedigree Construction and Genomic Breeding Value Evaluation**: DNA extracted from juvenile wing blood or feather samples provides genome-wide genotypes via DNA-chips or whole-genome sequencing (WGS). This genomic profile serves a dual purpose: it reconstructs precise pedigree matrices and calculates GEBVs simultaneously to finish genomic selection (GS), eliminating manual pedigree recording errors and simplifying field workflows (Huisman, 2017; Pértille, et al., 2016; Zhou, et al., 2024).

**(4) Conservation of Genetic Diversity and Environmental Adaptation**: The breeding population can be effectively integrated with GGP populations, giving the NMAW-PBS the potential for a much larger census size than conventional AI-based nucleus breeding programs. However, large census size alone does not guarantee a high effective population size: as detailed in the Disadvantages section, unequal reproductive success under natural mating can make realized *N_e_* substantially lower than census size and thereby accelerate inbreeding. The diversity advantage of large, open populations is therefore conditional rather than automatic and must be secured through active genomic management. Open, large-population breeding environments allow natural selection pressures to operate alongside artificial selection (Gao, et al., 2023; Restoux, et al., 2022). This synergy enhances the expression of superior variation combinations and promotes robust environmental adaptability.

**(5) System Alignment with High-Welfare Production Standards:** System design explicitly modeled around alternative housing configurations to match real-world, high-welfare commercial production environments. This system eliminates labor-intensive artificial insemination, which is also incompatible with animal welfare standards. Previous studies have also demonstrated that a balance between welfare outcomes and economic benefits of free-range farms can be obtained under optimized management conditions (He, et al., 2022).

### Disadvantages and technical constraints

**(1) High Initial Capital Investment:** Deploying high-throughput automated equipment capable of logging individual metrics (e.g., feed intake, body weight) in loose-housed birds demands significant initial capital expenditure (Lyu, et al., 2023; Peng, et al., 2022). Additionally, several phenotyping technologies remain in development and require further engineering. Complex diagnostic measurements, such as computed tomography (CT) scans, cannot be easily executed on unconstrained, freely moving birds, meaning some traits still require periodic manual intervention (Kou, et al., 2024).

**(2) Enterprise Integration Obstacles:** The proposed methodology departs fundamentally from established industrial protocols centered on battery cages and manual artificial insemination. Commercial breeding enterprises must execute extensive re-engineering of physical facilities, automation workflows, and staff training to shift away from traditional rooster-group natural mating designs or artificial insemination cages. Implementing a robust genomic selection pipeline introduces substantial ongoing laboratory and bioinformatic costs (Lourenco, et al., 2015; Wolc, 2015). Financial constraints currently limit many small-to-mid-sized breeding companies to traditional pedigree-based selection (PBLUP), slowing down industry-wide technological adoption.

**(3) Behavioral and Epidemiological Risks:** Uncontrolled natural mating eliminates precise mate allocation, which may heighten inter-bird aggression (flock fighting), and accelerate the horizontal transmission of infectious pathogens within the flock (Gao, et al., 2023; Restoux, et al., 2022; Xue, et al., 2021).

**(4) Data Collection Pipeline Risks:** The large-scale collection, transmission, and centralized storage of genomic data, individual phenotypic records, and behavioral profiles within an IoT-integrated breeding system introduce significant data security. As all information wholly depends on the data recording, transferring and storage, any data loss in any of the data collection time point might have serious issues for final individual genomic selection. Robust cybersecurity protocols, including end-to-end encryption, blockchain-based data integrity verification, and role-based access control, are essential to mitigate these risks (Alahe, et al., 2024).

**(5) Potential genetic risks under unrestricted natural mating:** Importantly, genomic mating and optimal contribution selection are particularly valuable in free-range natural mating systems because pedigree control through fixed mating pairs is largely absent. In practice, natural mating often results in unequal reproductive success among males due to social dominance, mate competition, and behavioral hierarchy, leading to reproductive skew and substantial variation in family size (Leimar and Bshary, 2022). Consequently, the realized effective population size (*N_e_*) may be considerably smaller than the census population size, thereby accelerating the accumulation of inbreeding and reducing long-term selection response (Meuwissen, 1997; Woolliams, et al., 2015). Recent advances in genomic mating and optimal contribution selection (OCS) provide effective approaches for balancing short-term genetic gain with long-term maintenance of genetic diversity by simultaneously optimizing individual genetic merit and genomic relationships among breeding candidates (Meuwissen, et al., 2020; Sonesson and Meuwissen, 2000). By optimizing parental contributions and mate allocation based on realized genomic relationships rather than expected pedigree relationships, these approaches effectively constrain the rate of inbreeding while maintaining high selection intensity and long-term genetic gain, thereby overcoming one of the principal limitations of unrestricted natural mating (Sonesson, et al., 2012; Woolliams, et al., 2015; Zhang, et al., 2022). Accordingly, the proposed breeding framework should integrate continuous genomic monitoring of effective population size, genomic relationship matrices, runs of homozygosity (ROH), and optimal contribution algorithms into routine breeding decisions to do more accurate control of inbreeding while maximizing the expected breeding values of future offspring. Such integration would preserve the welfare and management advantages of natural mating while minimizing inbreeding accumulation, maintaining long-term genetic diversity, and ensuring sustainable genetic improvement in commercial free-range poultry breeding populations (Meuwissen, et al., 2020; Woolliams, et al., 2015).

## Progress in related poultry breeding technologies

The successful implementation of the proposed framework critically depends on the maturity of several enabling technologies. Genomic breeding technologies have fundamentally reshaped selection efficiency across livestock industries (Wolc, 2015), while traditional manual scale measurements are rapidly being replaced by intelligent, non-contact digital monitoring systems (Li, et al., 2020; Reaño, et al., 2025). Operating a fully free-range, natural mating system requires high-throughput methods to capture phenotypes simultaneously, and genotypes timely. In this section, we briefly review the current state of automated phenotyping, pedigree reconstruction, genomic selection, and PLF infrastructure technologies that underpin the core technologies of our proposed system.

### Automated phenotyping technologies

Phenotypic evaluation in poultry involves a complex matrix of traits ranging from basic physical indicators (live body weight, feather cover, skeletal soundness) to intricate physiological performance data (feed efficiency, reproductive longevity, meat quality). These traits can be categorized into real-time continuous collection (e.g., feed intake, oviposition) and periodic point-in-time collection (e.g., body weight, carcass yield). While major proprietary breeding entities possess advanced automated devices, limited commercial availability has restricted widespread farm adoption. Fortunately, many end-point traits (egg quality parameters, structural skeletal health) can utilize existing semi-automated laboratory tools, though field-level instrumentation requires continued refinement.

**Body Weight and Feed Consumption:** Accurate, individual feed conversion ratio (FCR) tracking is fundamental to modern selection indexes. Automated feeding and weighing stations have successfully transitioned to commercial application for both layer and broiler lines (Lyu, et al., 2023; Peng, et al., 2022; Zhou, et al., 2023). Recent development in computer vision and deep learning allow for non-contact, real-time mass estimation using 3D point-cloud reconstructions from depth cameras (e.g., Intel RealSense, Microsoft Kinect) alongside multi-view RGB configurations. Artificial intelligence-driven contactless weighing platforms can monitor multiple birds simultaneously at high frequencies. These systems enable continuous refinement of predictive growth curves with <5% mean absolute percentage error (MAPE) relative to manual weighing (Lyu, et al., 2023; Reaño, et al., 2025). These methods are crucial for free-range systems where physical capture induces severe stress. Given that rapid-growth lines (e.g., modern broilers and commercial ducks) can achieve daily weight gains of 100 to 120 g, high-frequency, non-contact monitoring is vital for this framework.

However, free-range environments pose unique challenges to sensor reliability, including dust accumulation on optical devices, temperature-induced sensor drift, physical damage from animal contact, and variable lighting conditions under outdoor paddock settings. Regular calibration protocols (recommended at minimum weekly intervals), protective housing designs with IP65-rated enclosures, and adaptive image-processing algorithms capable of handling variable illumination are essential to maintain data quality under field conditions (Li, Ren, Li and Zeng, 2020; Neethirajan, 2022).

**(2) Automated Egg Production Recording:** Because the framework eliminates traditional battery cages, recording individual hen performance in a loose-housed environment is a primary challenge. Implementing automated smart nesting boxes equipped with RFID sensors is essential to log individual oviposition events, egg weight, and nesting behavior without compromising cage-free movement.

Physiological and Welfare Traits: Traits linked directly to animal welfare–such as gait mechanics, thermal profiles, and feather coverage–are becoming central to multi-trait selection breeding goals. Integrating artificial intelligence, machine vision, and near-infrared spectroscopy facilitates automated, high-precision measurement of these parameters (Milosevic, et al., 2019; Mohammadabadi, et al., 2024). Furthermore, medical imaging adaptations, specifically CT and 3D imaging, offer unprecedented precision in evaluating live birds. Computed tomography allows for non-invasive, highly accurate predictions of breast muscle yield, skeletal integrity, and total body composition, establishing comprehensive anatomical profiles from live selection candidates (Kou, et al., 2024).

### Large-scale pedigree construction and inbreeding control

Resolving parentage in chickens is uniquely challenging due to their oviparous biology and highly polygamous mating behavior (Huisman, 2017; Zhou, et al., 2024) when housed in unconstrained floor systems. Because this framework intentionally removes manual mating controls and artificial insemination, complex half-sib and full-sib family structures emerge naturally within the flock. Consequently, computational pedigree reconstruction is mandatory to calculate relationship matrices and avoid severe inbreeding depression.

For commercial‑scale implementation, based on recent empirical studies in poultry, one feasible cost-accuracy balancing example is using a minimum panel of approximately 700 markers filtered with MAF ≥ 0.05, which can achieve >95% parentage assignment accuracy using a 0.48 IBD threshold in the tested study populations (Yan, et al., 2025; Zhou, et al., 2024). These parameters are population-specific examples rather than universal fixed requirements; assignment performance is affected by candidate-parent pool size, background relatedness, reproductive skew, genotyping error and marker polymorphism. Higher-density panels (e.g., 10K–60 K SNP chips) may be warranted for populations with complex multi-generational structures or when full genomic selection pipelines are simultaneously deployed. Downstream bioinformatic algorithms–including exclusion-based methods, genomic relationship matrices (GRM), and likelihood-based software applications (e.g., COLONY)–have proven highly accurate in reconstructing complex multigenerational pedigrees from large, mixed-sex commercial cohorts (Huisman, 2017; Yan, et al., 2025; Zhou, et al., 2024).

Parentage mis-assignment will introduce noise into genomic relationship matrix, reduce GEBV accuracy, and distort estimation of coancestry and inbreeding rate, thereby weakening OCS inbreeding control. Therefore, in practical workflows, post-QC filtering steps are required: suspicious parent-offspring pairs with IBD values close to threshold should be flagged; higher-density markers can be supplemented for ambiguous individuals. Quantifying the tolerance threshold of parentage error for downstream genomic evaluation is an important future research topic for NMAW-PBS.

Furthermore, genomic relationship matrices capture Mendelian sampling and the realized sharing of chromosomal segments among individuals, enabling more precise management of genetic diversity than conventional pedigree-based methods and providing a robust foundation for sustainable breeding under large-scale free-range production systems (Meuwissen, et al., 2020; VanRaden, 2008).

### Genomic selection and environmental modeling

Genomic selection represents a milestone paradigm shift in quantitative animal genetics. The proposed framework replaces traditional pedigree-based PBLUP and phenotypic mass selection entirely with GEBV-driven selection indices. In commercial layer breeding, GS has accelerated genetic gain for egg numbers by over 30% (Wolc, et al., 2015), while simultaneously driving gains in FCR and growth velocity for meat-type broilers and ducks. Through these efficiencies, leading breeding operations have successfully compressed generation intervals from approximately 13 months down to 7 months in layers (Wolc, 2015).

As the cost of high-throughput sequencing declines, the integration of multi-dimensional data becomes a computational reality. Free-range systems expose birds to fluctuating climatic variables (temperature, relative humidity, stocking density), making the modeling of Gene-by-Environment (GxE) interactions imperative. For example, studies in native tropical chicken lines have revealed substantial antagonistic genetic correlations between production traits and thermal tolerance, proving that selection for extreme production under controlled conditions compromises thermal resilience due to high metabolic heat loads (Fathi, et al., 2022; Juiputta, et al., 2023).

To address GxE interactions within this framework, we advocate a multi-pronged statistical strategy. First, reaction norm models (also known as character-state models) should be implemented to estimate individual breeding values as a function of environmental gradients (e.g., ambient temperature, humidity index), effectively modeling each genotype’s phenotypic response across environmental clines (Kolmodin, et al., 2002). Second, multi-trait random regression models enable the estimation of breeding values across multiple environments simultaneously, allowing selection candidates to be ranked by their aggregated performance across representative environmental conditions rather than a single standardized test environment. Third, ssGBLUP models incorporating environmental covariates as fixed or random effects can partition additive genetic variance into components attributable to macro-environmental (barn-level) and micro-environmental (individual-level) variation. To balance production efficiency with climate resilience, selection indexes should apply economically weighted penalties on heat-susceptibility indicators (e.g., rectal temperature elevation, panting score, feed intake depression under thermal challenge) while maintaining positive selection pressure on core production traits. The framework specifically recommends deploying multi-environment testing (MET) protocols where selection candidates are distributed across at least two contrasting environmental stations (e.g., thermoneutral vs. heat-challenged), with genomic information capturing additive genetic effects common to both environments and reaction norms capturing genotype-specific environmental sensitivities (Pitesky, et al., 2020).

To counter production-resilience antagonisms, the framework deploys machine learning architectures (such as random forests and deep neural networks) to merge genomic markers with localized environmental sensor data (Mohammadabadi, et al., 2024; Pitesky, et al., 2020). By incorporating environmental covariates directly into ssGBLUP or reaction norm models, breeders can optimize multi-trait selection indexes that preserve production metrics while selecting for superior climate resilience and disease resistance (Ahmed, et al., 2024; Astill, et al., 2020).

Finally, it should be emphasized that the NMAW-PBS does not prescribe a single, fixed breeding objective. Breeding objectives and selection-index weights necessarily differ among breeding companies, species, and market positioning—for example, egg versus meat production, conventional versus welfare-certified premium markets, or temperate versus tropical target environments—and must therefore be defined by each breeding program individually. What the framework provides is the enabling infrastructure for any such objective: automated phenotyping supplies continuous individual records for production, reproductive (including traits expressed only under natural mating, such as fertility and mating behavior), welfare, behavioral, health, and climate-resilience traits; genomic evaluation and reaction-norm models convert these records into environment-specific breeding values; and established index methodology—bio-economic models, desired-gains approaches, constrained selection to manage antagonistic genetic correlations, and independent culling levels for minimum welfare standards—can then be applied, with objectives and weights revisited periodically as markets, regulations, and genetic (co)variances evolve.

### Precision livestock farming infrastructure

To successfully operate this framework, modern PLF systems must utilize a robust, three-layered IoT architecture.

(1) **Perception Layer:** This foundational layer integrates diverse sensor modalities for continuous data acquisition. Individual identification relies primarily on passive ultra-high-frequency RFID tags attached to wing bands or leg tags, supporting simultaneous multi-tag detection. Behavioral monitoring deploys micro-accelerometers for activity classification (feeding, resting, walking, dust-bathing) and early lameness detection. Environmental sensing encompasses temperature, relative humidity, ammonia (electrochemical sensors), and CO_2_ across the housing facility. Machine vision arrays employ IP67-rated RGB-D cameras to maximize coverage and minimize occlusion in loose-housed environments.
(2) **Network Layer:** Data transmission from free-range facilities requires low-power, long-range wireless communication protocols. The proposed framework can use different communication technologies for different tasks. NarrowBand-Internet of Things (NB-IoT) is a standards-based low power wide area technology, and can provide backup communication where gateway coverage is limited, and industrial Wi-Fi or wired Ethernet can be used for high-bandwidth video transmission from 3D camera systems.
(3) **Application Layer:** Cloud-based and edge-computing platforms process incoming data streams through automated analytics pipelines. A typical deployment for a 10,000-bird commercial free-range facility generates approximately 50–100 GB of raw sensor data daily (predominantly from video streams), requiring scalable cloud storage with automated tiering. As the volume of genomic data and high-frequency phenotypic time-series grows, the breeding infrastructure must be supported by high-performance data storage systems and optimized bioinformatic pipelines to manage the computational demands of large-scale genomic selection. This sensor-fusion approach allows for automated, early detection of disease anomalies, continuous welfare auditing, and immediate behavioral assessments (Rowe, et al., 2019). Edge computing handles localized real-time interventions, while digital twins simulate environmental impacts on flock dynamics. While digital twin technology holds promise for environmental impact simulation and predictive flock management, its application in poultry breeding remains in the early development stages, requiring extensive historical data for model calibration and validation (Peladarinos, et al., 2023). Current implementations are limited to simplified agent-based models of bird movement and resource utilization, with full genomic-phenotypic integration representing a significant future research direction.

## Future development and applications

Although the proposed free-range and natural mating breeding framework presents considerable potential for future commercial poultry breeding, substantial research is still required before its large-scale implementation. In particular, the economic feasibility of breeding systems that simultaneously optimize genetic gain, production efficiency, animal welfare, and environmental robustness remains largely unknown. For example, if substantially larger numbers of selection candidates are genotyped or whole-genome sequenced to enable accurate pedigree reconstruction and genomic selection, the cost-effectiveness of increased genomic investment must be carefully balanced against the expected genetic gain, phenotyping capacity, and infrastructure costs. Furthermore, the optimal breeding population size, mating group structure, and operational management strategies for different poultry species, including layers, broilers, ducks, and other avian species, remain to be systematically evaluated under commercial production conditions.

Genome editing in poultry has advanced rapidly over the past several years, transitioning from proof-of-concept studies in fibroblast cell lines to germline-competent editing in primordial germ cells (PGCs) and the generation of disease-resistant commercial chickens (He, et al., 2025; Sid and Schusser, 2018). CRISPR/Cas9 remains the dominant platform, with key milestones including precise editing of the ANP32A gene to confer resistance to avian influenza viruses (Idoko-Akoh, et al., 2023) and chNHE1 knockout to achieve complete resistance to ALV-J (Koslová, et al., 2020). Sex chromosome-targeted knock-in strategies have been optimized for efficient integration at chicken sex-linked loci (Cao, et al., 2026; Jung, et al., 2026). Looking forward, the streamline the production of genetically edited poultry, offering transformative opportunities for enhancing disease resilience, production efficiency, and animal welfare in commercial breeding programs.

The novel breeding system proposed in this study demonstrates potential significant advantages in accelerating the genetic transition from breeding populations to commercial tiers, ensuring strict animal welfare compliance, maintaining high genetic diversity, and increasing environmental adaptability. We stress that these outcomes are proposed benefits inferred from the structural properties of the framework, not empirically demonstrated advantages: their realization depends on successful management of reproductive skew, on accurate genomic pedigree reconstruction, and on the economics of automated phenotyping at scale, as discussed above.

The future of poultry breeding technology will evolve toward a precision breeding matrix integrating multiple automated technologies. The synergistic application of genomic selection, intelligent PLF networks, and reproductive adaptations achieves a revolutionary improvement in breeding efficiency (Astill, et al., 2020; Mohammadabadi, et al., 2024; Wolc, 2015). It enables a deep integration of breeding and farming through the dual optimization of “genetics + environment”. Through continuous optimization of the breeding architecture–balancing production metrics, animal welfare, and environmental sustainability–this framework provides solid support for the high-quality development of the global poultry industry.

This framework provides a conceptual foundation and actionable technical roadmap supporting potential high-quality development for global poultry industry. It is important to acknowledge that the compression of the breeding pyramid proposed herein necessitates concurrent adjustments in biosecurity protocols, and supply chain governance, aspects that require industry-wide stakeholder engagement beyond the technical scope of this paper. The transition from established multi-tier breeding structures to an integrated framework represents not merely a technological shift but a systemic transformation involving regulatory, economic, and organizational dimensions that warrant dedicated future investigation.

## CRediT authorship contribution statement

**Zhuo-Cheng Hou:** Conceptualization, Drafting, Writing and Revision of manuscript, Project management, Final approval, and Accountability.

## Disclosures

The authors declare that they have no known competing financial interests or personal relationships that could have appeared to influence the work reported in this paper.
